# Chronic lung disease in HIV-infected children established on antiretroviral therapy

**DOI:** 10.1097/QAD.0000000000001249

**Published:** 2016-11-08

**Authors:** Jamie Rylance, Grace Mchugh, John Metcalfe, Hilda Mujuru, Kusum Nathoo, Stephanie Wilmore, Sarah Rowland-Jones, Edith Majonga, Katharina Kranzer, Rashida A Ferrand

**Affiliations:** a Liverpool School of Tropical Medicine, Liverpool, UK; b Biomedical Research and Training Institute, Harare, Zimbabwe; c University of California, San Francisco, San Francisco, California, USA; d University of Zimbabwe, Harare, Zimbabwe; e University of Oxford, Oxford; f London School of Hygiene and Tropical Medicine; g Royal Free Hospital, London, UK.

**Keywords:** antiretroviral therapy, chronic lung disease, HIV, lung function, sub-Saharan Africa

## Abstract

**Objective:**

Respiratory disease is a major cause of morbidity and mortality in HIV-infected children. Despite antiretroviral therapy (ART), children suffer chronic symptoms. We investigated symptom prevalence, lung function and exercise capacity among older children established on ART and an age-matched HIV-uninfected group.

**Design:**

A cross-sectional study in Zimbabwe of HIV-infected children aged 6–16 years receiving ART for over 6 months and HIV-uninfected children attending primary health clinics from the same area.

**Methods:**

Standardized questionnaire, spirometry, incremental shuttle walk testing, CD4^+^ cell count, HIV viral load and sputum culture for tuberculosis were performed.

**Results:**

A total of 202 HIV-infected and 150 uninfected participants (median age 11.1 years in each group) were recruited. Median age at HIV diagnosis and ART initiation was 5.5 (interquartile range 2.8–7.5) and 6.1 (interquartile range 3.6–8.4) years, respectively. Median CD4^+^ cell count was 726 cells/μl, and 79% had HIV viral load less than 400 copies/ml. Chronic respiratory symptoms were rare in HIV-uninfected children [*n* = 1 (0.7%)], but common in HIV-infected participants [51 (25%)], especially cough [30 (15%)] and dyspnoea [30 (15%)]. HIV-infected participants were more commonly previously treated for tuberculosis [76 (38%) vs 1 (0.7%), *P* < 0.001], had lower exercise capacity (mean incremental shuttle walk testing distance 771 vs 889 m, respectively, *P* < 0.001) and more frequently abnormal spirometry [43 (24.3%) vs 15 (11.5%), *P* = 0.003] compared with HIV-uninfected participants. HIV diagnosis at an older age was associated with lung function abnormality (*P* = 0.025). No participant tested positive for *Mycobacterium tuberculosis*.

**Conclusion:**

In children, despite ART, HIV is associated with significant respiratory symptoms and functional impairment. Understanding pathogenesis is key, as new treatment strategies are urgently required.

## Introduction

Respiratory disease is the most common manifestation of HIV/AIDS among children, accounting for more than 50% of HIV-associated mortality [1–4]. The use of antiretroviral therapy (ART) and co-trimoxazole prophylaxis has contributed to a reduction in the rate of acute respiratory tract infections and mortality among HIV-infected children in both high-resource and low-resource settings [5]. In the pre-ART era, 30–40% of HIV-infected children also developed chronic lung disease (CLD), most commonly due to lymphoid interstitial pneumonitis (LIP), but this condition responds well to ART and is now uncommon in clinical practice, except in children aged below 5 years [6–8].

Nevertheless, recent studies in Southern Africa have demonstrated that about 30% of African HIV-infected older children have chronic respiratory symptoms, classically a chronic cough [often leading to presumptive treatment for tuberculosis (TB)] and reduced exercise tolerance [3,4]. In these studies, even participants with pronounced respiratory impairment looked well at rest, not all had cough, and plain radiological abnormalities were subtle and not consistent with LIP [9]. However, these studies did not include HIV-uninfected controls and included a mix of children who were ART-naive as well as those taking ART. The aim of this study was to investigate the burden and features of CLD among HIV-infected children established on ART and in an age-matched HIV-uninfected group.

## Methods

The study was carried out between September 2014 and June 2015 at the Harare Children's Hospital HIV clinic in Harare, Zimbabwe – a public sector clinic that provides HIV care for more than 4000 children. Children were eligible for the study if they were aged between 6 and 16 years, had been taking ART for at least 6 months, were not acutely unwell and were not taking TB treatment. We consecutively recruited up to five eligible participants per day, restricted to this number due to logistical constraints. A comparison group of HIV-uninfected children in the same age group was also recruited from seven clinics that provided HIV testing and counselling to all attendees regardless of the reason for attendance and served the same population as that of Harare Hospital (high population density suburbs, with small dwellings being typical). We enrolled children who had tested HIV-negative and who were not acutely unwell and who were not receiving treatment for respiratory infection or TB. A sample size of 200 HIV-1-infected children was selected to provide a precision of ±6% around an estimated 25% prevalence of CLD, defined as any one of unexplained chronic cough for at least 3 months; hypoxaemia (SpO_2_ < 90%) at rest or desaturation on exertion by more than 4% from baseline; abnormal spirometry without another medical explanation, for example asthma; and chronic dyspnoea [Medical Research Council (MRC) breathlessness score >1] [10].

### Data collection

A nurse-administered questionnaire was used to collect details of socio-demographic indices, clinical history and current symptoms. A standardized examination was performed including WHO staging of HIV infection, measurement of height and weight, spirometry and exercise testing.

Spirometry was performed according to American Thoracic Society (ATS) standards using EasyOne World spirometers (ndd Medical Technologies, Inc., Andover, Massachusetts, USA) [11]. Up to eight forced exhalations were recorded while sitting. We analysed only data from individuals who produced three consistent traces that met ATS quality criteria. The highest forced expiratory volume in 1 s (FEV_1_) and forced vital capacity (FVC) measurements for each individual were used, with other indices recorded from the best trace (largest total of FEV_1_ and FVC). Obstruction was defined as a reduced FEV_1_ : FVC. For clarity, we use the term ‘reduced FVC’ in which FVC was low with a normal FEV_1_ : FVC. We avoided the term ‘restriction’ as we were unable to measure lung volumes. In the cases in which any spirometric abnormality was found, participants underwent repeat spirometry 15 min after administration of 2.5 mg nebulized salbutamol. An improvement in FEV_1_ of at least 12% was considered to represent significant reversibility.

An incremental shuttle walk test (ISWT) was performed as a measure of cardiorespiratory fitness. This was devised for adults [12], but has been validated for use in children with chronic respiratory disease [13]. Participants were not tested if, at rest, SpO_2_ was less than 88%, heart rate (HR) was more than 110 and respiratory rate was more than 30 breaths/min. On flat ground, participants were instructed to walk between two markers placed 10 m apart. A prerecorded series of ‘bleeps’ were played, which determined how quickly each 10 m segment should be completed. The standardized protocol demanded that the participant walked 30 m in the first minute, with each subsequent minute escalating the distance by 10 m (i.e. 40, 50 and 60 m in the second, third and fourth minutes, respectively). The test was terminated when participants were unable to reach the next marker by the time the beep was issued. The respiratory rate, HR and oxygen saturations were measured before and immediately after the end of the test. Only the first 82 participants in the HIV-uninfected group underwent ISWT due to limited staffing. Predicted maximal HRs were calculated by the Tanaka equation – 208 − 0.7 × age [14].

### Laboratory investigations

Where possible, all HIV-positive participants had sputum samples obtained by spontaneous expectoration or by induction using nebulized hypertonic saline. Sputum smears were examined by Ziehl–Neelson stain microscopy and a single mycobacterial culture was performed on Lowenstein–Jensen media. Sputum from HIV-uninfected participants was sought only if the WHO TB symptom screen was positive [15]. HIV viral load was measured with COBAS Ampliprep/Taqman 48 Version 2.0 (Roche, Rotkreuz, Switzerland) and CD4^+^ cell count was measured using an Alere PIMA CD4^+^ (Waltham, Massachusetts, USA) machine (HIV-positive participants only).

### Data analysis

Data were extracted from paper forms using optical character recognition software (Cardiff TELEFORM Intelligent Character, Version 10.7; Hewlett Packard, Palo Alto, California, USA). Data analysis was carried out using Stata v12 (StatCorp, College Station, Texas, USA) and GraphPad Prism v6 (GraphPad, La Jolla, California, USA). Height-for-age and BMI-for-age *z*-scores were calculated using the WHO reference standards [16]. Normal spirometric ranges were defined using the GLI Global Lung Initiative 2012 equation that determines race-specific and sex-specific reference values, taking account of height and age [17]. The lower limit of normal is defined as 1.64 SDs below the mean expected value (which describes the lowest 10 centiles as ‘abnormal’).

Shuttle walk and CD4^+^ results were treated as parametric data, with Student's *t* test used to compare means between HIV-infected and noninfected groups. Other continuous variables were nonparametric: central tendency was reported by the median and interquartile range (IQR), using the Mann–Whitney *U* test for equivalence testing between groups. Frequencies of categorical data – symptoms, past medical complaints and rates of abnormal spirometry and growth indices – were compared between HIV-infected and noninfected by chi-squared test. Results were considered statistically significant at *P* less than 0.05. The association of abnormal lung function with *a priori* defined clinical data was investigated using univariable logistic regression, and reporting odds ratios (ORs) with a Wald 95% confidence interval (CI). Stepwise backward multivariate logistic regression was then used; this incorporated age and sex, and those variables with individual *P* less than 0.1 on univariate testing.

Ethical approval was granted by the Medical Research Council of Zimbabwe, the Harare Hospital Ethics Committee, the Biomedical Research and Training Institute Institutional Review Board and the London School of Hygiene and Tropical Medicine Ethics Committee. All guardians gave written consent, and participants gave assent to participate in the study.

## Results

### Participant characteristics

**Fig. 1:**
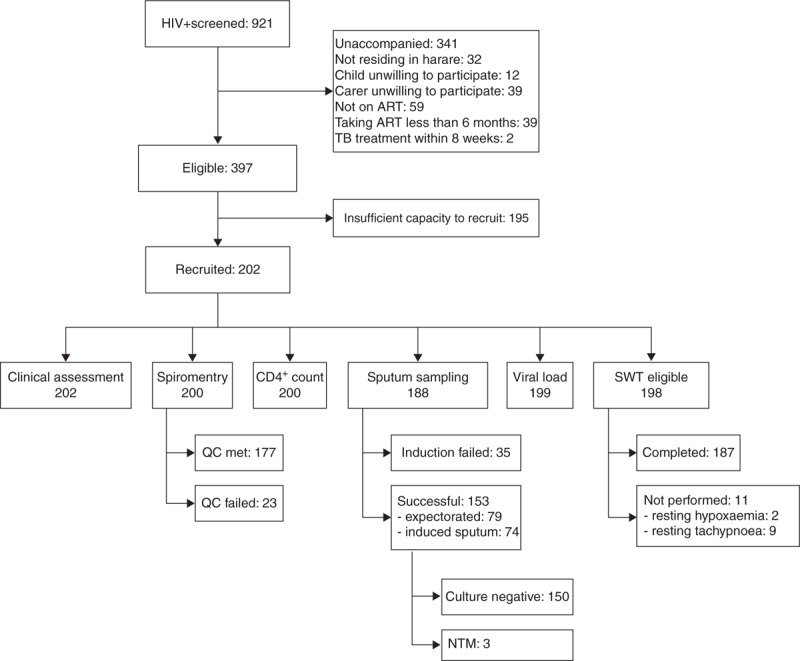
Flow chart of participant recruitment and testing.

A total of 202 HIV-infected participants were recruited: median age 11.1 (IQR 9.0–12.9) years and 55% men. Summary statistics are given in Table 1, and a flow diagram of participation and testing is given in Fig. 1. Of the 150 HIV-uninfected participants recruited as a comparison group, 42% were men and the median age was 11.0 (IQR 9.0–13.9) years. All but one HIV-infected participants were vertically infected, and the median age at HIV diagnosis was 5.5 (IQR 2.8–7.5) years (Table 2). The median duration of ART was 4.7 (IQR 2.6–6.4) years, with 161 (80%) taking nonnucleoside reverse transcriptase inhibitor–based (first-line) ART and the remainder taking a protease inhibitor–based regimen (Table 3). The median CD4^+^ cell count at HIV diagnosis (available for 105 participants) was 353 (IQR 134–696) cells/μl, and the CD4^+^ cell count at enrolment was 726 (IQR 476–941) cells/μl. The majority of participants (79%) had an HIV viral load less than 400 copies/ml (Table 1), and 194 (96%) were taking co-trimoxazole.

Diagram showing recruitment of HIV-infected participants. Four participants did not attend for follow-up appointment and therefore did not undergo SWT. NTM, nontuberculous mycobacteria.

None of the HIV-uninfected participants screened positive using the WHO TB symptom screen. Within the HIV-infected group, 153 participants produced samples for mycobacterial culture, 35 could not despite sputum induction and 16 were too ill or did not return for the procedure. None grew *Mycobacterium tuberculosis*, but three grew nontuberculous mycobacteria (NTM) (not speciated).

A significantly higher proportion of HIV-infected participants were stunted than HIV-uninfected participants (35.8 vs 7.3%, *P* < 0.001), but prevalence of wasting was similar in the two groups (Table 1). Seventy-six HIV-infected children had been previously treated for TB, compared with one from the HIV-uninfected group.

The proportion treated for asthma was similar in both groups. Chronic respiratory symptoms (breathlessness, cough or wheeze) were reported by 25.3% of HIV-infected children but only by one (0.7%) HIV-uninfected child. Wheeze was infrequently reported in either group (Table 1).

Incremental shuttle walk test (ISWT) could not be performed in 15 HIV-infected participants due to resting hypoxaemia (*n* = 2), resting dyspnoea (*n* = 9) or loss to follow-up (*n* = 4). One HIV-uninfected participant had tachypnoea at rest. The distance attained during ISWT was significantly reduced in the HIV-infected group [mean 771 m (SD 216) compared with 889 m (SD 227) in the HIV-uninfected group, *P* < 0.001]. Shortly after completion of the test, mean HR was not significantly difference at 62 (SD 12.0) and 67% (SD 10.5) of their predicted maximal HRs for the HIV infected and noninfected groups, respectively.

**Fig. 2:**
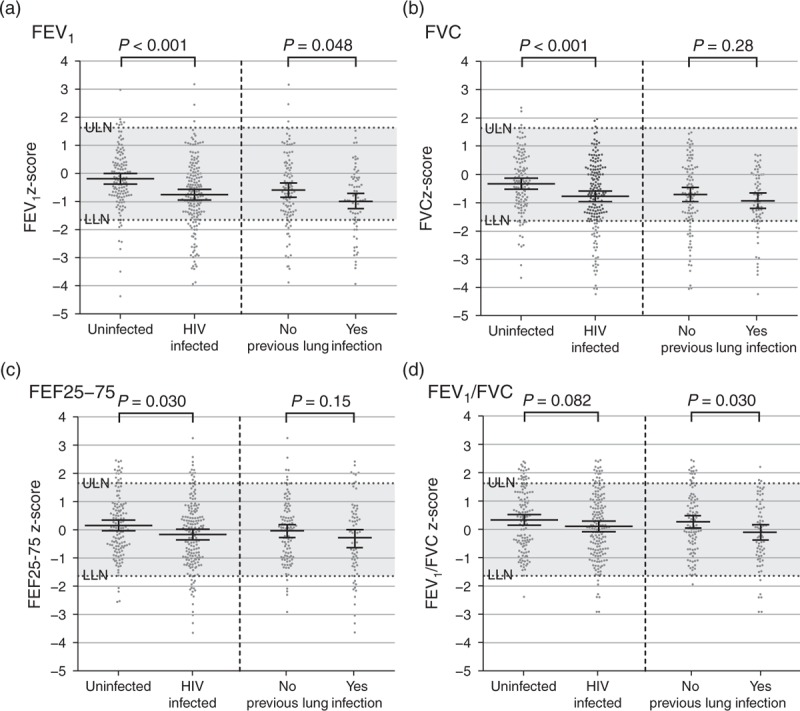
Spirometric abnormalities in HIV-infected participants and HIV-uninfected participants.

One hundred and seventy-seven (88%) HIV-infected and 130 (87%) HIV-uninfected participants had high-quality spirometry traces. Quality grading according to ATS standards did not differ between groups either before or after reversibility testing. Of those high-quality (interpretable) traces, a quarter of all HIV-infected participants had abnormal lung function on spirometry. This was significantly higher than among HIV-uninfected participants (24.3 vs 11.5%, *P* = 0.01). This was reflected in lower FEV_1_, FVC and FEF25–75 indices in the HIV-infected group (*P* < 0.05) (Fig. 2). There was no significant difference in the FEV_1_ : FVC ratio (*P* = 0.08) between the two groups, although those with a history of lung infection had a lower FEV_1_ : FVC than those without (*P* = 0.03). For those with spirometric abnormality, postbronchodilator reversibility traces were complete and adequate in 31 HIV-infected and six HIV-uninfected participants, demonstrating reversibility in 11 (35.4%) and two (33.33%), respectively.

Dot plots of all participants with spirometry results, presented as *z*-scores compared with GLI 2012 reference ranges. Bars illustrate the mean and SDs of each group. Dotted horizontal bars show the limits of normality. *P* values are derived from *t* tests. HIV-infected individuals are further shown by stratification by ‘Prior lung infection’. This is a compound definition, including individuals with any of tuberculosis, PCP, nontuberculous mycobacteria by sputum culture, and hospitalization for chest infection.

Predictors of abnormal lung function are summarized in Table 4. When subdivided into restrictive and obstructive types, numerators were insufficient to draw robust conclusions: data are therefore presented as ‘normal’ or ‘abnormal’. The presence of any respiratory symptom, except wheeze, was significantly associated with abnormal lung function. Older age at HIV diagnosis was positively associated with abnormal lung function (*P* = 0.025), as was wasting (OR 8.1, 95% CI 2.3–31.7). After fitting a multivariate model, wasting was the only independent predictor: OR 4.7 (95% CI 1.2–18.6).

We investigated the potential for prior respiratory infection to impact on lung function by stratified analysis (Fig. 2 and Supplemental Table S1). Among those with previous infection (any of TB, Pneumocystis pneumonia, NTM by sputum culture and hospitalization for chest infection), those with later presentation and ART initiation were more likely to have abnormal lung function (*P* < 0.05). Dyspnoea, tachypnoea, oxygen desaturation and wasting were also disproportionately apparent (*P* < 0.05 for each). However, among those without previous infection, cough and sputum production were more commonly seen in those with abnormal spirometry (*P* < 0.05).

## Discussion

A quarter of HIV-infected children experienced chronic respiratory symptoms, despite being treated with ART and with good virological control in the majority. In contrast, only one HIV-uninfected participant reported respiratory symptoms. More than one-third of the HIV-infected children in our study had been previously treated for TB, although we were unable to determine how the diagnosis of TB had been made due to a lack of robust records. As noted in other cohorts, where background TB rates are high, overtreatment is likely in those with chronic respiratory symptoms due to a lack of diagnostic and alternative therapeutic strategies for chronic respiratory symptoms [18,19]. Notably, no TB was found in our study on microbiological investigation.

We found much higher rates of abnormal lung function among HIV-infected compared with HIV-uninfected children. Development of airways disease in early life has been described in the context of HIV infection: airway resistance measurements indicate that abnormalities of airway calibre are established in those as young as 1 year old [20]. However, in resource-limited settings, no longitudinal studies have been conducted to define how such abnormalities change over time or how they respond to ART. Interestingly, in our current study, obstructive lung disease was less common than in a study conducted in Malawi (4 vs 26%) [4]. In the Malawi study, however, 31% of individuals were ART-naive or had been taking ART for a relatively shorter duration (median 20 months) [2].

In the pre-ART era, the most common cause of CLD was LIP found in 30–40% of HIV-infected children [7,21]. LIP responds well to ART and the prevalence has significantly declined with increased availability of ART [22]. We found low rates of wheeze and bronchodilator response, which would suggest that the chronic respiratory symptoms are not likely to be due to asthma. Similarly, although household air pollution is detrimental to lung function and can cause these symptoms, rates of biomass as the main fuel for cooking or lighting are lower in our cohort (less than one in five) than other reported periurban series in sub-Saharan Africa, due to access to mains electricity [23].

In the ART era, multiple aetiological factors may contribute to lung disease, including long-term sequelae of repeated bacterial and viral respiratory tract infections and possibly HIV-induced chronic inflammation and immune senescence caused by dysregulated immune activation [24,25]. Such processes account for the high prevalence of bronchiectasis in this population and may also contribute to obliterative bronchiolitis [18,21]. The latter has been described in a previous study from Zimbabwe. It seems to represent, as in non-HIV-related obliterative bronchiolitis, a final common pathway of lung injury typified radiologically by air trapping and mosaic attenuation of the lung parenchyma [2,9]. The features on plain chest radiography are subtle and nonspecific, and high-resolution computed tomography is the definitive imaging modality. The lack of high resolution computed tomography (HRCT) facilities may explain why obliterative bronchiolitis is not recognized in Africa, despite the high prevalence of chronic respiratory symptoms [26–29]. Clinically, obliterative bronchiolitis usually presents as an obstructive lung disease with minimal response to inhaled beta agonists. However, the pattern of spirometry abnormalities observed in our study was mainly restrictive. This might be explained by chronic immune activation causing scarring and fibrosis, resulting in a restrictive lung function abnormality [30–32]. We found a reduced FEV_1_/FVC ratio – a more obstructive pattern – in those with a previous history of infection. This would be consistent with unrecognized obliterative bronchiolitis that has been treated as TB or other infection. However, TB in adults is itself known to leave evidence of airway obstruction, even in those cured of disease.

Our finding that later diagnosis is associated with abnormal spirometry suggests that early diagnosis and treatment of HIV may prevent development or progression of CLD. Established disease appears to persist despite otherwise effective ART. This was apparent in children with a history of infection, in which later initiation of ART and signs of reduced gas-exchange capacity (oxygen desaturation and tachypnoea) predicted lung function abnormality. Wasting, also a predictor of lung function abnormality, could be represented by association among many risk factors for lung disease, including nutrition and chronic inflammatory disease.

In our cohort, there was no significant correlation between lung function abnormality and SWT distance. ISWT is a valid and reproducible predictor of aerobic exercise capacity (VO_2_ peak). As a maximal test, it is less variable than self-paced protocols such as the 6-min walk test [13]. VO_2_ peak is determined by both respiratory and cardiovascular fitness. It is possible that the reduced exercise capacity in HIV-infected children might be partly due to cardiac disease. Previously noted echocardiographic abnormalities in Zimbabwean children include left ventricle hypertrophy and diastolic dysfunction seen in 74 of 110 (67%) and 27 of 110 (24%), respectively [33]. We are further investigating this possibility using echocardiography in our cohort.

The strengths of this study was that it was prospectively conducted, recruitment was unselective (i.e. not based on presence of respiratory symptoms) and from a general HIV clinic. It included an HIV-uninfected comparison group recruited from the same geographical area. Study limitations include self-report of illness, which may result in recall bias. Spirometry was not available for all participants due to exclusion of poor quality traces and ISWT was not universally performed in HIV-uninfected participants due to staff shortages. Furthermore, we were unable to measure lung volumes. Our HIV-uninfected group were not matched by sex, resulting in females being slightly more frequently represented compared with in the HIV-infected group. However, this should not affect interpretation for spirometry data as lung function indices use sex-specific normal ranges. For exercise capacity, male children tend to outperform females, but subanalysis of our data stratified by sex revealed no difference in findings (data not shown) [34].

Our study demonstrates that there is a large burden of chronic respiratory morbidity among HIV-infected children established on ART. Despite efforts to eliminate mother-to-child transmission, 300 000 infants were newly infected with HIV in 2011, and a record number (630 000) were receiving ART in low-income countries [35,36]. Thus, HIV will continue to place a heavy burden on paediatric clinical services in sub-Saharan Africa, where 90% of the world's HIV-infected children live [37]. Delay in diagnosis of HIV infection and initiation of ART may increase the risk of developing CLD. The WHO until 2015 had recommended that children aged over 5 years should be treated with ART once CD4^+^ cell count drops to 500 cells/μl or Stage 3 or 4 disease. The recent START and TEMPRANO trials in adults demonstrated that treating HIV infection irrespective of immunological status reduces the risk of AIDS and non-AIDS events [38,39]. In children, earlier HIV recognition may reduce the risk of developing CLD by preventing recurrent respiratory tract infection and by reducing chronic pulmonary inflammation. Adoption of universal treatment of all HIV-infected individuals regardless of disease stage may therefore be advantageous, and some additional benefit might be expected from co-trimoxazole prophylaxis by lowering the rates of lower respiratory tract infection. However, once established, CLD appears to persist despite ART. Hitherto, the main focus of HIV programmes has been on meeting the need for massive scale-up of paediatric ART and on improving infant survival. There is now a pressing need to address the long-term complications of HIV infection among the increasing numbers of children growing up with HIV, and novel diagnostic and therapeutic strategies for CLD are urgently required.

## Acknowledgements

We would like to thank the clinic staff and the participants and their families.

Funding: The study was funded by the Nina Ireland Program for Lung Health and the Wellcome Trust (R.A.F., grant number 095878/Z/11/Z).

Contributions: Study conception: R.A.F., J.R., K.K. and J.M.; study design: R.A.F., J.R., K.K. and J.M.; protocol development: R.A.F., J.R., K.K. and J.M.; training and quality control: J.R.; data collection: G.M., R.A.F., E.M. and SW; data analysis: J.R., R.A.F. and K.K.; drafting and revision of manuscript: all authors.

### Conflicts of interest

There are no conflicts of interest.

## Supplementary Material

Supplemental Digital Content

## Figures and Tables

**Table 1:** Summary characteristics of study participants by HIV status.

	HIV uninfected (*n* = 150)	HIV infected (*n* = 202)	*P* value
Age, median years (IQR)	11.1 (9.0–12.8)	11.1 (9.0–12.9)	0.77
Sex, female, *n* (%)	63 (42.0)	111 (55.0)	0.018
Orphan, *n* (%)	20 (13.5)	102 (51.0)	<0.001
Mother known to be HIV infected	13 (8.7)	202 (100)	<0.001
Active smoker, *n* (%)	0 (0)	0 (0)	–
Passive smoke exposure at home, *n* (%)	27 (18.0)	42 (20.9)	0.18
Any respiratory complaint^a^, *n* (%)	1 (0.7)	51 (25.3)	<0.001
Dyspnoea (MRC grade >1), *n* (%)	0 (0)	30 (14.9)	<0.001
Daily cough for >1 month, *n* (%)	1 (0.7)	30 (14.9)	<0.001
Sputum production, *n* (%)	1 (0.7)	20 (10.0)	<0.001
Wheeze, *n* (%)	0 (0)	9 (4.5)	0.007
Resting tachypnoea: rate >25, *n* (%)	9 (6.0)	28 (14.1)	0.016
Hospital admission for RTI in last year, *n* (%)	3 (2.0)	4 (2.0)	0.96
Antibiotics for RTI in last year, *n* (%)	3 (2.0)	45 (22.3)	<0.001
Previously diagnosed or treated			
Asthma, *n* (%)	3 (2.0)	7 (3.5)	0.37
PCP, *n* (%)	0 (0)	6 (3.0)	0.029
TB ever, *n* (%)	1 (0.7)	76 (37.8)	<0.001
TB more than once, *n* (%)	0 (0)	5 (2.5)	0.10
Stunted (HFA *z*-score <2), *n* (%)	12 (7.9)	72 (35.8)	<0.001
Wasting (BFA *z*-score <2), *n* (%)	10 (6.7)	18 (9.0)	0.42
ISWT	(*n* = 82)	(*n* = 187)	
Desaturates during test, *n* (%)	5 (6.1)	22 (11.1)	0.16
ISWT distance, metres mean (SD)	889 (227)	771 (216)	<0.001
Spirometry interpretation	(*n* = 130)	(*n* = 177)	
Normal, *n* (%)	115 (88.5)	134 (75.7)	0.003
Obstruction, *n* (%)	1 (0.8)	7 (4.0)	0.052
Reduced FVC, *n* (%)	14 (10.8)	36 (20.3)	0.012
Bronchodilator response	(*n* = 6)	(*n* = 31)	
Reversibility demonstrated, *n* (%)	2 (33.3)	11 (35.5)	0.92

Missing data on two participants. BFA, body mass for age; HFA, height for age; IQR, interquartile range; ISWT, incremental shuttle walk test; MRC, Medical Research Council; PCP, Pneumocystis pneumonia; RTI, respiratory tract infection; TB, tuberculosis.

^a^Wheeze, chronic cough or dyspnoea.

**Table 2:** HIV-specific summary characteristics.

	HIV infected (*n* = 202)
Age at diagnosis, median (IQR)	5.5 (2.8–7.5)
Age at ART initiation, median (IQR)	6.1 (3.6–8.4)
Mode of HIV transmission, *n* (%)	
Mother to child	201 (99.5)
Sexual	1 (0.5)
Reason for HIV testing	
Chronic cough	113 (55.9)
Hospital admission	41 (20.3)
Repeated illness	32 (15.8)
Other^a^	16 (7.9)
CD4^+^ at diagnosis, median (IQR)^b^	353 (134–696)
CD4^+^, median (IQR) at recruitment	726 (476–941)
HIV viral load <400 copies/ml, *n* (%)	155 (78.7)

ART, antiretroviral therapy; IQR, interquartile range; TB, tuberculosis.

^a^Other reasons were testing in elective male circumcision (*n* = 6), spontaneous healthcare worker initiated (*n* = 2), TB diagnosed (*n* = 1), sexual debut prompted testing (*n* = 1) and don’t recall (*n* = 6).

^b^Data available for 105 participants from health records.

**Table 3:** HIV treatment regimens.

	NNRTI based		PI based	
	NVP	EFV	ATAZ/r	LPV/r
ZDV/3TC^a^	72	23	7	2
TDF/3TC	32	32	25	–
ABC/3TC	–	1	2	–
ABC/DDI	–	1	2	1
Total	161		39	

3TC, stavudine; ABC, abacavir; ATAZ/r, ritonavir-boosted atazanavir; DDI, didanosine; EFV, efavirenz; LPV/r, ritonavir-boosted lopinavir; NVP, nevirapine; TDF, tenofovir; ZDV, zidovudine.

^a^One individual unknown NNRTI or PI. One individual DDI/ATAZ/r with second NRTI unrecorded.

**Table 4:** Association of factors with abnormal lung function in HIV-infected children.

	Normal (*n* = 134)	Abnormal (*n* = 43)	OR (95% CI)
Age at diagnosis, *n* (IQR)	4.6 (2.7–6.7)	5.8 (3.8–8.8)	1.15 (1.02–1.29)^*^
Age at ART initiation, *n* (IQR)	5.8 (3.5–8.3)	6.3 (4.3–8.8)	1.05 (0.95–1.17)
Years on ART, *n* (IQR)	4.6 (2.3–6.4)	5.1 (3.4–6.4)	1.08 (0.95–1.23)
Any symptom, *n* (%)	25 (18.7)	20 (46.5)	3.8 (1.7–8.5)^*^
Dyspnoea, *n* (%)	13 (9.7)	14 (32.6)	4.5 (1.7–11.5)^*^
Daily cough, *n* (%)	12 (9.0)	13 (30.2)	4.4 (1.7–11.7)^*^
Sputum production, *n* (%)	7 (5.2)	9 (20.9)	4.8 (1.5–16.2)^*^
Wheeze, *n* (%)	6 (4.5)	3 (7.0)	1.6 (0.2–7.9)
Passive smoker	33 (24.6)	9 (20.9)	0.8 (0.3–2.0)
Biomass fuel used for cooking	21 (15.7)	8 (18.6)	1.2 (0.4–3.2)
Biomass fuel or candles used for lighting	18 (13.4)	6 (14.0)	1.0 (0.3–3.0)
Previous TB treatment, *n* (%)	49 (36.6)	18 (41.9)	1.2 (0.6–2.7)
Stunting (HFA < −2), *n* (%)	45 (33.6)	19 (44.2)	1.6 (0.7–3.3)
Wasting (BFA < −2), *n* (%)	5 (3.7)	10 (3.8)	8.1 (2.3–31.7)^*^
Abnormal SpO_2_ at rest or exercise, *n* (%)	11 (8.5)	9 (23.1)	3.2 (1.1–9.4)^*^
Resting tachypnoea (rate >25), *n* (%)	16 (12.1)	8 (19.1)	1.7 (0.6–4.7)
Viral load suppressed <400 copies/ml^a^	105 (80.2)	32 (78.1)	0.9 (0.4–2.4)
ISWT distance, metres (SD)^b^	765 (212)	771 (241)	1.00 (1.00–1.00)
CD4^+^ at recruitmentc^c^ (SD)	774 (342)	688 (344)	1.00 (1.00–1.00)

ART, antiretroviral therapy; CI, confidence interval; ISWT, incremental shuttle walk test; IQR, interquartile range; OR, odds ratio; TB, tuberculosis.

^*^Significant at *P* < 0.05 by Mann-Whitney U-test (for non-parametric data) and Student t test (for parametric data).

^a^*n* = 131 and 41, respectively.

^b^*n* = 131 and 38, respectively.

^c^*n* = 134 and 42, respectively.
