# Supplementary material for: Chronic lung disease in HIV-infected children established on antiretroviral therapy
Source: AIDS. 2016 Nov 8;30(18):2795–803. doi: 10.1097/QAD.0000000000001249 (PMC5106089; doi:10.1097/QAD.0000000000001249)
Supplement: Supplemental Digital Content [file aids-30-2795-s001.docx]

# Supplementary Table S1

Stratified analysis of the associations of pre-selected clinical and physiological characteristics with abnormal lung function.

|  | **No prior infection (n=105)** | | | **Prior infection (n=72)** | | |
| --- | --- | --- | --- | --- | --- | --- |
|  | **Normal**  **n=82** | **Abnormal**  **n=23** | **OR (95% CI)** | **Normal**  **n=52** | **Abnormal**  **n=20** | **OR (95% CI)** |
| Age at diagnosis†, n (IQR) | 4.6 (2.2 – 6.7) | 4.4 (3.7 – 7.5) | 1.05 (0.90 – 1.23) | 4.6 (3.3 – 6.7) | 7.9 (5.0 – 9.0) | 1.30 (1.07 – 1.59)* |
| Age at ART initiation†, n (IQR) | 6.1 (3.2 – 8.4) | 5.4 (3.7 – 7.9) | 0.96 (0.83 – 1.10) | 5.3 (3.6 – 8.3) | 8.0 (5.7 – 9.9) | 1.19 (1.00 – 1.41)* |
| Years on ART, n (IQR) | 4.3 (2.1 – 6.4) | 5.4 (3.7 – 6.7) | 1.05 (1.00 – 1.42)* | 4.6 (3.0 – 6.5) | 4.5 (2.7 – 6.4) | 0.94 (0.76 – 1.15) |
| Any symptom, n (%) | 14 (17) | 7 (30) | 2.1 (0.7 – 6.1) | 11 (21) | 13 (65) | 6.9 (2.2 – 21.5)* |
| Dyspnoea, n (%) | 9 (11) | 5 (22) | 2.3 (0.7 – 7.5) | 4 (8) | 9 (45) | 9.8 (2.6 – 37.8)* |
| Daily cough, n (%) | 4 (5) | 6 (26) | 6.9 (1.7 – 27.1)* | 8 (15) | 7 (35) | 3.0 (0.9 – 9.7) |
| Sputum production, n (%) | 2 (2) | 4 (17) | 8.4 (1.4 – 49.3)* | 5 (10) | 5 (25) | 3.1 (0.8 – 12.3) |
| Wheeze, n (%) | 4 (5) | 1 (4) | 0.9 (0.1 – 8.3) | 2 (4) | 2 (10) | 2.8 (.04 – 21.2) |
| Passive smoker, n (%) | 22 (27) | 6 (26) | 1.0 (0.3 – 2.8) | 11 (21) | 3 (15) | 0.7 (0.2 – 2.6) |
| Biomass fuel used for cooking, n (%) | 12 (15) | 5 (22) | 1.6 (0.5 – 5.2) | 9 (17) | 3 (15) | 0.8 (0.2 – 3.5) |
| Biomass fuel or candles used for lighting, n (%) | 11 (13) | 3 (13) | 1.0 (0.2 – 3.9) | 7 (13) | 3 (15) | 1.1 (0.3 – 4.9) |
| Stunting (HFA<-2), n (%) | 22 (27) | 7 (30) | 1.2 (0.4 – 3.3) | 23 (44) | 12 (60) | 1.9 (0.7 – 5.4) |
| Wasting (BFA <-2), n (%) | 3 (4) | 3 (13) | 4.0 (0.7 – 21.2) | 2 (4) | 7 (37) | 14.6 (2.7 – 79.3)* |
| Abnormal SpO_2_ at rest or exercise, n (%) | 7 (9) | 4 (19) | 2.5 (0.6 – 9.3) | 4 (8) | 5 (28) | 4.4 (1.0 – 18.9)* |
| Resting tachypnoea (rate >25), n (%) | 13 (16) | 3 (14) | 0.8 (0.2 – 3.2) | 3 (6) | 5 (25) | 5.3 (1.1 – 25.0)* |
| Viral load suppressed <400 copies/ml^a^ | 60 (75) | 18 (86) | 2.0 (0.5 – 7.5) | 45 (88) | 14 (70) | 0.3 (0.1 – 1.1) |
| ISWT distance, metres (SD)^b^ | 803 (223) | 804 (254) | 1.00 (1.00 – 1.00) | 704 (180) | 730 (223) | 1.00 (1.00 – 1.00) |
| CD4 at recruitment^c^, cells/µl (SD) | 761 (343) | 658 (259) | 1.00 (1.00 – 1.00) | 794 (342) | 721 (423) | 1.00 (1.00 – 1.00) |

“Prior infection” uses a compound definition of any of the following: previous TB treatment; previous PCP treatment; admitted to hospital with infection in the preceding 12 months; mycobacteria isolated in sputum culture

a: No prior infection - normal n=80, abnormal n=21; Prior infection - normal n=51, abnormal n=20

b: No prior infection - normal n=80, abnormal n=21; Prior infection - normal n=51, abnormal n=17

c: No prior infection - normal n=82, abnormal n=22; Prior infection - normal n=52, abnormal n=20

* significant at p<0.05
